# The Cost-Effectiveness of 13-Valent Pneumococcal Conjugate Vaccine in Seven Chinese Cities

**DOI:** 10.3390/vaccines9111368

**Published:** 2021-11-20

**Authors:** Yan Li, Huaqing Wang, Wesley Furnback, Bruce C. M. Wang, Shuiqing Zhu, Peng Dong

**Affiliations:** 1 Chinese Center for Disease Control and Prevention, Beijing 100050, China; liyan2@chinacdc.cn (Y.L.); wanghq@chinacdc.cn (H.W.); 2 Elysia Group, LLC, 199 Water St 14th Floor, New York, NY 10038, USA; wes.furnback@elysiagroup.com (W.F.); bruce.wang@elysiagroup.com (B.C.M.W.); 3 Pfizer Investment Co., Ltd., Shanghai 200041, China; peng.dong@pfizer.com

**Keywords:** cost-effectiveness, pneumococcal conjugate vaccine, PCV 13, China

## Abstract

**Objective:** This study estimates the cost-effectiveness of vaccination with the 13-valent pneumococcal conjugate vaccine (PCV13) among infants in Beijing, Shanghai, Shenzhen, Chengdu, Karamay, Qingdao, and Suzhou. **Methods:** A previously published cost-effectiveness model comparing vaccination with PCV13 to no vaccination was localized to the included Chinese cities. A systematic literature review was undertaken to identify age-specific incidence rates for pneumococcal bacteremia, pneumococcal meningitis, pneumonia, and otitis media (AOM). Age-specific direct medical costs of treating the included pneumococcal diseases were taken from the Chinese Health Insurance Association database. The base case analysis evaluated vaccine efficacy using direct effect and indirect effects (DE+ IDE). A subsequent scenario analysis evaluated the model outcomes if only DE was considered. A vaccination rate of 70% was used. The model reported outcomes over a one-year period after it was assumed the vaccine effects had reached a steady state (5–7 years after vaccine introduction) to include the direct and indirect effects of vaccination. Health outcomes were discounted at 5% during the steady-state period. **Results:** Vaccination with PCV13 was cost-effective in the base case analysis for all included cities with the incremental cost-effectiveness ratio (ICER) ranging from 1145 CNY(Shenzhen) to 15,422 CNY (Qingdao) per quality-adjusted life-year (QALY) gained. PCV13 was the dominant strategy in Shanghai with lower incremental costs and higher incremental QALYs. PCV13 remained cost-effective in the DE-only analysis with all ICERs falling below a cost-effectiveness threshold of three times GDP per capita in each city. **Conclusions:** Vaccination with PCV13 was a cost-effective strategy in the analyzed cities for both the DE-only and DE + IDE analyses. PCV13 became very cost-effective when a vaccination rate was reached where IDE is observed.

## 1. Introduction

Invasive pneumococcal diseases (IPD) caused by *Streptococcus pneumoniae* (*S. pneumonia*) include meningitis and bacteremia. *S. pneumonia* can also cause non-invasive pneumococcal infections such as pneumonia and acute otitis media (AOM). The World Health Organization (WHO) has estimated 1 million children die from pneumococcal disease each year, with the majority in developing countries [1]. Patients with pneumococcal diseases also experience decreased quality of life and incur significant direct and indirect costs due to medical healthcare resource utilization, absenteeism, and presenteeism [2].

The burden of pneumococcal diseases in China is significant, with an estimated 12% of global cases occurring in China [3]. In China lower respiratory infections including pneumonia were the 12th leading cause of overall deaths and premature deaths (years of life lost) in 2017 [4]. Pneumococcal diseases pose a significant burden in China where there are an estimated 30,000 deaths per year [3,5]. In 2000, it was estimated there were 22 million cases of pneumonia among children younger than five years of age in China [6]. Of patients dying of pneumonia, it is estimated that nearly 50% are attributable to *S. pneumonia* [7].

Pneumococcal conjugate vaccines (PCVs) have been proven to be safe and effective interventions to prevent pneumococcal diseases. The first PCV available on the market was Prevenar 7^®^, a 7-valent pneumococcal conjugate vaccine (PCV7) in the United States (U.S.), which was introduced in February 2000. PCV7 protects against seven pneumococcal serotypes (4, 6B, 9V, 14, 18C, 19F, and 23F), Prevenar 13^®^, a 13-valent pneumococcal conjugate vaccine (PCV13), which was introduced to the U.S. market in 2010, protects against thirteen pneumococcal serotypes (1, 3, 4, 5, 6A, 6B, 7F, 9V, 14, 19A, 19F, 18C, and 23F) starting at two months of age. PCVs (including PCV13 and PCV10) are recommended to be included in all childhood immunization programs according to the WHO [8].

The introduction of PCV7 and subsequently PCV13 has led to significant declines in invasive pneumococcal disease, pneumonia, and otitis media in settings with universal vaccination programs [9,10,11,12,13]. The effectiveness of vaccination has been felt through both the direct effect and through the indirect effect of vaccination [14].

The immunogenicity and safety of PCV13 in infants was studied in a Phase 3 clinical trial conducted in China [15]. The double-blind study compared PCV13 with PCV7 among two or more month old infants in China [15]. The study showed PCV13 was non-inferior to PCV7 among the seven common serotypes, and PCV13 offered additional protection and immune response to the six additional serotypes without any additional safety concerns [15]. Prevenar 13^®^ was licensed in China in 2016 and is approved for a 3 + 1 dosing schedule. This analysis considers cost and effectiveness data specifically for Prevenar 13^®^, which have been derived from the literature. Several PCV13 vaccinations have been launched in China but due to differences in the production technique and ingredients, further studies are needed to provide more context to their long-term safety and effectiveness compared to Prevenar 13^®^.

PCV13 has been shown to be cost-effective compared to the standard of care (either no vaccination or PCV7) in different global settings [16]. In China, there have been four studies published examining the cost-effectiveness of PCV13 [17,18,19,20]. All four studies compared PCV to no vaccination, and in all cases, PCV13 was cost-effective using a cost-effectiveness threshold of 3× GDP per capita. However, there have not been any studies assessing the cost-effectiveness of PCV13 in specific Chinese cities. As vaccine funding in China is based on cities, this analysis selected a sample of cities ranging from first- to fifth-tier with varying socioeconomic and geographic characteristics.

The objective of this study was to estimate the cost-effectiveness of 70% vaccination with PCV13 compared to no vaccination in Beijing, Shanghai, Shenzhen, Suzhou, Chengdu, Karamay, and Qingdao.

## 2. Methods

### 2.1. Approach

A previously published cost-effectiveness model assessing the cost-effectiveness of implementing a PCV13 program was localized to seven cities in China [19,21,22,23,24,25]. The seven cities included Beijing, Chengdu, Karamay, Qingdao, Shanghai, Shenzhen, and Suzhou. These cities were chosen according to their geographical distribution and as representative of the socioeconomics and healthcare patterns of their respective regions. The details of the model structure and design have been well published [19,21,22,23,24,25]. Patients (infants) first enter the model and are assigned vaccine protection based on the vaccine uptake, which was assumed to be 70% in this analysis.

There are a total of eight health states within the model, two each for invasive pneumococcal diseases (IPD), pneumonia (PNE), and acute otitis media (AOM), in addition to no illness and death. Within IPD there are the pneumococcal bacteremia and pneumococcal meningitis states, within PNE there are the inpatient and outpatient states, and within AOM there are the mild and moderate/severe states.

**Figure 1:**
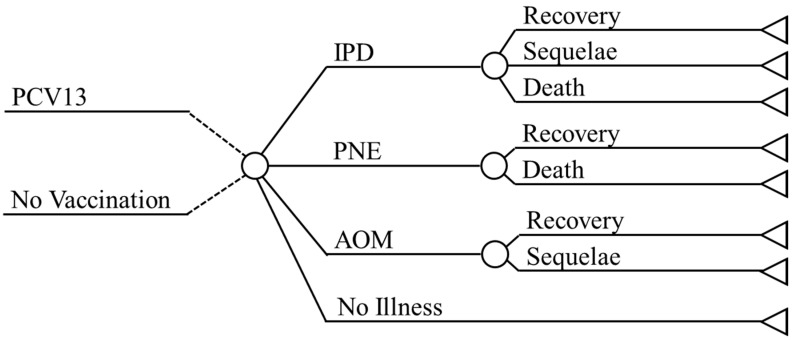
Model Design. Abbreviations: PCV = Pneumococcal conjugate vaccine; IPD = Invasive pneumococcal diseases; PNE = Pneumonia; AOM = Acute otitis media.

A decision analytic model (Figure 1) is used to determine the outcomes (recovery, sequelae, or death) associated with the IPD, PNE, and AOM health states.

The model took a payer perspective for each city, and outcomes were calculated over a one-year time horizon after a steady-state period was reached, which was assumed to take 5–7 years, to include the direct and indirect effects of vaccination. Outcomes were recorded for each arm in the model (vaccination and no vaccination) and included the number of cases and deaths for each health state, the number of quality-adjusted life-years lost due to death, sequelae, and acute disease, vaccination costs, and treatment-related costs of the included health states. Life-years were estimated over a lifetime time horizon, by using the conditional expected remaining life-years according to each age group in the model. Lifetime health outcomes were discounted at a rate of 5%, and costs were expressed in CNY. Per the approved vaccination schedule of Prevenar 13^®^ in China, children were to receive a 3 + 1 schedule, which included a total of 4 doses in the base case analysis. A separate analysis explored the impact of a 2 + 1 dosing schedule on the results of the base case analysis.

### 2.2. Demographic Inputs

Key demographic data was included for each of the analyzed cities in China and included the total population, the percentage of patients under one year of age, and the age distribution [26].

### 2.3. Epidemiologic Inputs

The model considers age-based incidence rates for pneumococcal meningitis, pneumococcal bacteremia, pneumonia (inpatient and outpatient), otitis media (mild and moderate/severe), and sequelae (Table 1). Incidence rates were calculated based on rates retrieved from a systematic literature review and the results from an analysis of the Chinese Health Insurance and Research Association Database (CHIRA) (Supplementary Materials: Table S1.). The CHIRA database includes data from a sample (2–10%) of Tier 1 municipalities, capital cities of each province (Tier 2), prefecture-level cities (Tier 3), and country-level cities (Tier 4). The methodology used to collect the data for CHIRA has been well described by Shen et al. [19].

To better understand the context of the incidence rates retrieved from the CHIRA database and compare them to others that have been used in China, a systematic literature review (SLR) was undertaken. The objective of the SLR was to identify economic evaluations of PCVs for mainland China between 1/1/2006 and 10/11/2016. Of the 1012 records identified there were seven studies of PCVs focusing on mainland China [17,18,19,20,27,28,29,33].

Each of the studies identified in the SLR was analyzed to extract the age-based incidence rates for bacteremia, meningitis, pneumonia, and otitis media, as well as the source(s) for the incidence rates. The rates and sources are shown in Supplementary Material Table S1.

Bacteremia and meningitis incidence rate sources included CHIRA [19], Taiwan’s Health Insurance Research Database (HIRD) [18,20], Chen et al. [27,28], and studies using surveillance data from the literature [17,29].

Incidence rates for inpatient pneumonia were identified from CHIRA [19], the HIRD [18,20], and studies using the literature [27,28]. Outpatient pneumonia incidence rates included CHIRA [19], and the HIRD [18,20]. Otitis media incidence rates for both mild and moderate/severe cases were identified in the CHIRA 2013–2015 database, the HIRD [18,20], and using the literature [19].

Within each of the diseases, age-based incidence rates were calculated for each source by taking the average across all studies using the data source. Point estimates for each disease were calculated by then taking an average across all of the sources. This method ensures that sources not weighted by their frequency of use in economic evaluations.

The results of the SLR and subsequent data extraction did find that CHIRA may overestimate the incidence of inpatient pneumonia. The point estimates for meningitis, bacteremia, pneumonia, and AOM developed through an average of all available sources were utilized in the model (Table 1).

The model also considered disease sequelae for cases of pneumococcal meningitis. Sequelae included neurological impairment (7.0%) and hearing loss (13.0%) [35].

Case fatality ratios for bacteremia, meningitis, and inpatient pneumonia and serotype coverage parameters were derived from Shen et al. [19]. Case fatality ranged 1–6% depending on age in bacteremia, 0–14% for meningitis, and 1–16% for inpatient pneumonia [17,33]. Pneumococcal bacteremia and pneumococcal meningitis were assumed to have 87.1% of cases caused by PCV13 serotypes [30]. Estimates of 92.3% and 81.5% were used for the serotype coverage of pneumonia and otitis media, respectively [31,32].

### 2.4. Vaccine Effectiveness

The direct and indirect effects of vaccination are both considered in this model. The derivation of the effectiveness rates for PCV13 was previously described in Shen et al., [14,19,34,35,36,37,38]. The direct effect of PCV13 refers to the reduction of disease in children that are vaccinated, while indirect effect refers to the herd effect or the reduction in disease for unvaccinated individuals. Herd effect within PCV13 has been well documented in the literature [14,37,38]. It has been estimated that the herd effect is observed with vaccination rates starting at 54% [14].

A scenario analysis examined a 2 + 1 vaccination schedule, which has been previously studied, and is suited for situations with high uptake in a national immunization program [39]; a 3 + 1 schedule is recommended in low uptake settings to maximize individual protection. A recent study comparing 3 + 1 and 2 + 1 vaccination schedules in China reported IgG geometric mean concentrations and OPA geometric mean titers trended numerically higher with 3 + 1 versus 2 + 1 dose schedules after the 3 priming doses versus 2 priming doses, respectively, in the first year of life [40]. However, no significant differences in immunogenicity were observed between the 3 + 1 versus 2 + 1 dose schedules after the toddler dose [40]. PCV13 was well-tolerated across all schedules [40]. Therefore, while direct protection in the first year of life may differ, we assumed 3 + 1 and 2 + 1 vaccination schedules had similar effectiveness when uptake in the infant population was greater than 70%.

### 2.5. Direct Costs

Direct treatment-related costs for pneumococcal meningitis, pneumococcal bacteremia, inpatient and outpatient pneumonia, and mild and moderate/severe otitis media were included in the model (Table 2). Costs were sourced from the CHIRA database, which provided costs for Tier 1 cities and national costs. In cases where data was not available specifically for Tier 1 cities, the national cost data was used. Treatment-related costs were assumed to include all of the direct medical costs for the treatment of a case and were specific to each age group.

A cost of 698 CNY was used for each dose of PCV13 for all included cities. Costs related to the service fee of the vaccination and transportation were only considered if they were direct costs. Service and transportation fees per dose, sourced from provincial governments or based on assumptions, were applied to Shanghai (5.5 CNY) (Assumption), Suzhou (20 CNY) [41], Shenzhen (25 CNY) [42], Beijing (25 CNY) [43], Chengdu (30 CNY) [44], and Qingdao (34 CNY) [45,46].

### 2.6. Quality of Life

Utility values were assigned to patients in the model to calculate the health-related quality of life. The general population of healthy patients was assumed to have a utility of 0.9. Disutility values through QALY decrements were applied to patients with bacteremia (0.008), meningitis (0.023), inpatient pneumonia (0.006), outpatient pneumonia (0.004), and acute otitis media (0.005) [47]. These QALY decrements were applied once for each observed case, which aimed to represent the short-term impact of the disease.

Patients with long-term sequelae were assigned separate utilities, which were applied to life-years to calculate QALYs. Neurological impairment and hearing loss due to pneumococcal meningitis carried utilities of 0.60 and 0.80, respectively [48].

### 2.7. Analysis

The base case analysis considered both DE and IDE and compared the costs and health outcomes of vaccination with PCV13 with no vaccination. For each arm of the model, the total number of QALYs and costs are reported and the incremental values for all outcomes between the PCV13 vaccination and no vaccination arms are calculated. The incremental cost-effectiveness ratio (ICER) is calculated by dividing the incremental total costs by incremental total QALYs to determine the cost per additional QALY for vaccination compared to no vaccination. China has not published an explicit cost-effectiveness threshold for the willingness to pay for an additional QALY. The China Guidelines for Pharmacoeconomic Evaluations, published in 2020, endorse the use of GDP per capita to calculate the willingness to payer threshold [49]. Interventions with ICERs less than GDP per capita income are considered “very cost-effective” and ICERs between 1–3x GDP per capita are considered “cost-effective”.

A one-way sensitivity analysis was performed by using the known confidence intervals of parameters or varying the parameters by +/− 10%. The minimum and maximum values of the disease incidence rates were used as the low/high values. Furthermore, an analysis of the incidence rates was undertaken to address the uncertainty within the point estimates of the incidence rates. For all included disease states the minimum and maximum values for each point estimate found either in the CHIRA database or the literature were recorded. A “minimum” and “maximum” scenario analysis were undertaken where all of the minimum values were used simultaneously in the minimum scenario and all of the maximum values were used in the maximum scenario.

Lastly, a scenario analysis evaluated the outcomes of the model when only DE was considered.

## 3. Results

### 3.1. Base Case Results

Table 3 shows the results of the base case analysis. Compared with no vaccination, PCV13 reduced the number of pneumococcal disease cases (thousands) by 46.33 (Beijing), 46.30 (Shanghai), 33.92 (Chengdu), 22.91 (Shenzhen), 21.72 (Qingdao), 21.30 (Suzhou), and 1.10 (Karamay). The reduction in pneumococcal disease cases resulted in QALYs saved (thousands) of 14.83 (Shanghai), 12.01 (Beijing), 9.71 (Chengdu), 7.66 (Shenzhen), 6.87 (Suzhou), 5.43 (Qingdao), and 0.27 (Karamay).

In Shanghai the cost of vaccination (295,111,028) was fully offset by savings in pneumococcal disease costs of 323,757,862 CNY, resulting in total cost savings of 28,646,835 CNY. In the other analyzed cities, the cost of vaccination was reduced by savings in pneumococcal disease-related costs, resulting in incremental total costs of 8764,793 CNY (Shenzhen), 4062,450 CNY (Karamay), 49,163,176 CNY (Beijing), 60,918,974 CNY (Suzhou), 83,713,944 CNY (Qingdao), and 113,549,270 CNY (Chengdu).

Vaccination with PCV13 dominated the no vaccination strategy in Shanghai with lower incremental costs and higher incremental QALYs. In the remaining cities, ICERs ranged from 1145 CNY in Shenzhen to 15,422 CNY in Qingdao. All ICERs were below 1× GDP per capita in each city.

The base case scenario was further analyzed by the total number of vaccination doses, a 2 + 1 (three total doses) vaccination schedule was evaluated along with the base case 3 + 1 (four total doses) (Table 4). Vaccination with PCV13 remained cost-effective across both vaccination schedules.

**Figure 2:**
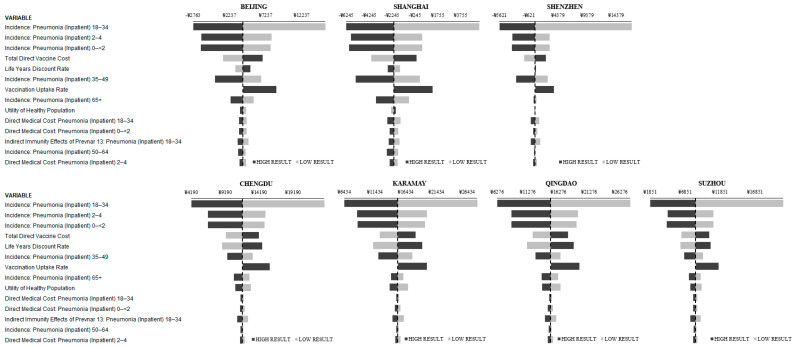
IDE + DE: One-Way Sensitivity Analysis Results (Tornado Diagrams).

One-way sensitivity analysis was performed on the base case (DE + IDE) analysis for each city (Figure 2). The top ten most influential parameters according to the spread between the high and low results, for each comparison and city were recorded. The incidence of inpatient pneumonia in those aged 18 to 34, 2 to 4, and 0 to less than 2 were the three most influential parameters. The total direct vaccine cost and the discount rate, which was varied between 3% and 7%, were the fourth and fifth most influential parameters, respectively.

### 3.2. Incidence Rate Analysis

**Figure 3:**
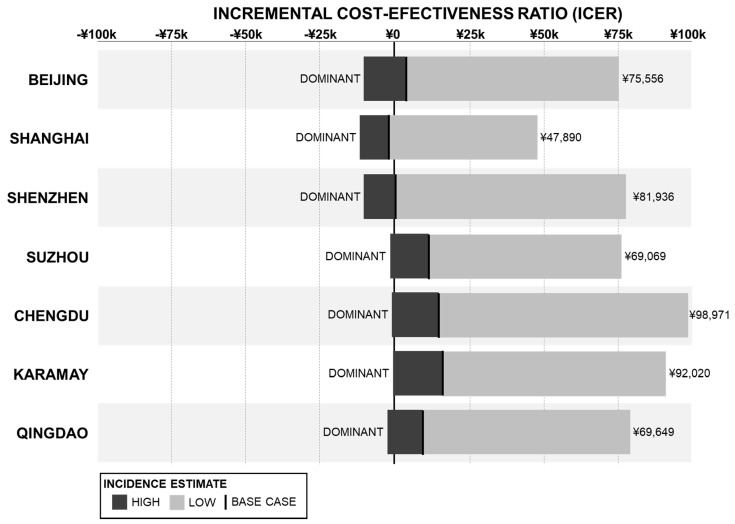
Scenario Analysis of Disease Incidence Rates—ICER Results.

The results for the scenario analysis comparing the minimum and maximum pneumococcal disease incidence rates for IPD, pneumonia, and otitis media are shown in Figure 3. Vaccination with PCV13 dominated the no vaccination strategy when the maximum incidence rate values were used and increased to between 47,890 CNY (Shanghai) and 98,971 CNY (Chengdu) when all the minimum incidence rate values were used, both remaining below the 1× GDP per capita threshold.

### 3.3. DE-Only Scenario

The results of the DE-only scenario analysis are shown in Table 5. The total number of pneumococcal disease cases (thousands) avoided with PCV13 vaccination was 26.03, 23.85, and 11.73 in the cities of Beijing, Shanghai, and Shenzhen, respectively. In the other analyzed cities, the number of pneumococcal disease cases (thousands) avoided was 0.64 in Karamay, 11.01 in Suzhou, 12.38 in Qingdao, and 18.41 in Chengdu. Vaccination with PCV13 provided higher incremental QALYs (thousands) of 1.59 (Beijing), 1.46 (Shanghai), 1.13 (Chengdu), 0.76 (Qingdao), 0.72 (Shenzhen), 0.67 (Suzhou), and 0.04 (Karamay) compared to no vaccination. Incremental total costs including direct medical and vaccination costs were higher for the PCV13 vaccination program compared to no-vaccination. The ICERs for vaccination with PCV13 were 161,237 CNY (Shenzhen), 155,625 CNY (Shanghai), 161,237 CNY (Beijing), 161,710 CNY (Karamay), 167,466 CNY (Suzhou), 170,345 CNY (Chengdu), and 171,496 CNY (Qingdao). All ICERs fell below the threshold of three times GDP per capita in the respective city.

## 4. Discussion

Vaccination with PCV13 at a rate of 70% was cost-effective in Beijing, Shanghai, Shenzhen, Suzhou, Chengdu, Karamay, and Qingdao. PCV13 remained cost-effective when a 2 + 1 dosing schedule was considered as well as in the separate sensitivity analysis of the disease incidence rates, when low incidence rate values were utilized, and became the dominant strategy with high incidence rate values. In the DE-only scenario, PCV13 was again cost-effective with all ICER falling below three times GDP per capita for each city. As vaccination with PCV13 was cost-effective in the DE-only and IDE + DE scenarios, it can be assumed to be cost-effective across all rates of vaccination, regardless of the presence of herd effect. While we assumed that a 3 + 1 and 2 + 1 schedule provide equivalent protection, it should be noted that a 3 + 1 schedule remains the preferable schedule where uptake of PCVs remains low [39,50]. Furthermore, while both schedules elicited equivalent immune responses following the booster dose, and we assumed similar protection, it has been hypothesized and modeled in the US that using two priming doses could result in incremental disease cases due to reduced protection in the first year of life, specifically when uptake is low and herd protection cannot be achieved [51].

The results of this study show that vaccination with PCV13 across all cities would avoid 2270 cases of IPD, 167,899 cases of pneumonia, and 23,407 cases of AOM in the base case analysis considering direct and indirect effects of vaccination. In the base case scenario, there were 3853 deaths avoided and an increase of 56,831 QALYs and 287,334,913 CNY in incremental total (vaccination and direct medical) costs.

Several other studies have assessed the cost-effectiveness of PCVs in China. PCV7 vs. no vaccination was examined by six studies [17,18,27,28,30,33], PCV10 vs. no vaccination by one [17], and PCV13 vs. no vaccination by four [17,18,19,20].

All four studies evaluating PCV13 vs. no vaccination found PCV13 to be cost-effective. The ICERs for PCV13 vs. no vaccination were 79,304 CNY (direct effect only), 76,551 CNY (direct effect and indirect effect for pneumonia only), and 3777 CNY (direct and indirect effect) in the study by Shen et al. These outcomes are similar to the ICERs in this analysis. Maurer et al. reported an ICER of 11,464 USD (CNY not reported), Mo et al., an ICER of 29,460 USD (182,652 CNY), and Zhou et al., 20,709 USD (140,821 CNY) for direct effect only and 18,483 USD (125,684 CNY) for direct and indirect effect. All of these ICERs fell below the threshold of 3× the current GDP per capita.

Incidence rates were highly influential parameters on the results of this study, and on the results of the cost-effectiveness studies in the literature. Studies using CHIRA data had significantly higher rates of inpatient pneumonia than those using HIRD data, local surveillance data, or other sources. Rather than focus on a single data source this study took an average of the point estimates for each source. Additionally, the low and high values observed in the literature for each point estimate were tested in the incidence rate analysis. While disease incidence did significantly influence the ICER for each city, PCV13 remained cost-effective when the low incidence rates were tested, and became the dominant strategy when the high incidence rates were tested.

The direct costs related to the vaccine and age distributions were the only parameters that varied from city to city, as all other parameters, including disease incidence rates, were consistent. As expected, lower direct vaccine and vaccine-related costs resulted in lower ICERs. Cities with younger populations tended to have lower ICERs as the vaccine’s effectiveness was higher in these younger populations. However, age distribution was only influential on the results in the base case scenario, which considered the indirect effects of the vaccine.

This study has several limitations. First, serotype coverage was assumed to be the same across all included cities. Large countries such as China can have considerable epidemiological differences between cities, especially those such as Karamay, with a smaller and demographically unique population. The model also did not consider serotype replacement in the long run. Second, the estimates of disease incidence vary considerably depending on the source for all included health states, especially inpatient pneumonia. These incidence rates are highly influential on the outcomes of the model as made evident by the one-way sensitivity analysis. Third, treatment costs were assumed across all tier 1 and non-tier 1 cities in the analysis. Costs are likely to vary from city to city within tier 1 and non-tier 1 cities. Lastly, the model may underestimate the benefit of vaccination with PCV as the number of patients carrying vaccine-serotype *Streptococcus pneumoniae* [52] and the prevalence of antimicrobial resistance [53] may decrease.

## 5. Conclusions

Vaccination with PCV13 is a cost-effective strategy for both the IDE + DE and DE-only scenarios in Beijing, Shanghai, Shenzhen, Suzhou, Chengdu, Karamay, and Qingdao. At vaccination rate levels where IDE are observed, PCV13 becomes very cost-effective with ICERs falling below GDP per capita in the respective cities. PCV13 remained cost-effective regardless of the dosing schedule and through an extended analysis of disease incidence. We estimate that vaccination with PCV13 will prevent a considerable number of cases of IPD, pneumonia, and AOM, as well as avoid mortality associated with IPD and pneumonia for both the vaccinated and unvaccinated population through the direct and indirect effect of the vaccine. This analysis can be used by policymakers when deciding on the allocation of resources in the studied cities.

## Figures and Tables

**Table 1:** Clinical Model Inputs.

Incidence (per 100,000)	0–2	2–4	5–17	18–34	35–49	50–64	65+
Pneumococcal Bacteremia † [17,18,19,20,27,28,29]	5.03 (1.40–10.20)	5.75 (1.98–9.47)	16.64 (0.48–48.81)	4.81 (0.54–12.48)	4.50 (0.24–12.59)	3.54 (0.50–9.21)	14.57 (0.57–36.00)
Pneumococcal Meningitis † [17,18,19,20,27,28,29]	2.49 (0.65–5.10)	1.59 (0.31–4.73)	5.14 (0.24–14.59)	1.43 (0.12–3.83)	1.41 (0.08–4.02)	1.20 (0.09–3.41)	2.37 (0.06–6.88)
Pneumonia							
Inpatient † [18,19,20,27,28]	8969 (2949–19,719)	8740 (2681–19,710)	4675 (334–13,027)	788 (110–2116)	497 (111–1190)	656 (305–1221)	2661 (1856–3700)
Outpatient † [18,19,20]	12,797 (1568–24,027)	15,749 (1642–29,857)	3644 (643–6646)	771 (186–1355)	662 (166–1158)	1035 (138–1931)	3405 (147–6663)
Otitis Media							
Mild † [18,19,20]	9147 (2.32–16,762)	8746 (29.45–15,531)	2002 (24.87–3978)	626 (5.39–1247)	767 (5.89–1528)	948 (3.17–1892)	1043 (1.63–2085)
Moderate to Severe † [18,19,20]	2793 (15.53–4472)	2650 (105–3953)	494 (98.43–889)	72.16 (10.62–134)	130 (8.39–252)	174 (4.99–343)	182 (2.19–361)
† CHIRA 2013–2015							
**Pre-PCV13 Serotype Coverage**	**Base Case**						
Pneumococcal Bacteremia [30]	0.87						
Pneumococcal Meningitis [30]	0.87						
Pneumonia							
Inpatient [31]	0.92						
Outpatient [31]	0.92						
Otitis Media							
Mild [32]	0.82						
Moderate to Severe [32]	0.82						
**Case Fatality Ratio**	**0–2**	**2–4**	**5–17**	**18–34**	**35–49**	**50–64**	**65+**
Pneumococcal Bacteremia [17,33]	0.04	0.01	0.05	0.05	0.06	0.06	0.06
Pneumococcal Meningitis [17,33]	0.14	0.03	0.04	0.00	0.12	0.00	0.02
Inpatient Pneumonia [17,33]	0.01	0.01	0.01	0.06	0.06	0.01	0.16
**Direct Effects**	**0–2**	**2–4**					
Pneumococcal Bacteremia [34]	0.94	0.94					
Pneumococcal Meningitis [34]	0.94	0.94					
Pneumonia							
Inpatient [34,35]	0.29	0.29					
Outpatient [34,35]	0.07	0.07					
Otitis Media							
Mild [36]	0.08	0.08					
Moderate to Severe [36]	0.17	0.17					
**INDIRECT EFFECT**	**0–2**	**2–4**	**5–17**	**18–34**	**35–49**	**50–64**	**65+**
Pneumococcal Bacteremia [14,37,38]	0.64	0.64	0.53	0.32	0.32	0.18	0.12
Pneumococcal Meningitis [14,37,38]	0.64	0.64	0.53	0.32	0.32	0.18	0.12
Inpatient Pneumonia [14,37,38]	0.22	0.17	0.00	0.12	0.05	0.02	0.03

**Table 2:** Cost Inputs.

Direct Medical Costs	0–2	2–4	5–17	18–34	35–49	50–64	65+
Pneumococcal Bacteremia							
Tier 1	¥695	¥2450	¥542	¥408	¥901	¥1407	¥2147
National	¥695	¥2450	¥542	¥408	¥901	¥1407	¥2147
Pneumococcal Meningitis							
Tier 1	¥377	¥87	¥321	¥3131	¥356	¥4640	¥13,587
National	¥25,602	¥5315	¥6967	¥13,105	¥5988	¥9345	¥6427
Pneumonia							
Inpatient							
Tier 1	¥5470	¥3942	¥6030	¥11,372	¥29,034	¥17,567	¥31,486
National	¥4618	¥3101	¥3348	¥5140	¥5090	¥7518	¥11,820
Outpatient							
Tier 1	¥232	¥233	¥271	¥433	¥538	¥566	¥639
National	¥195	¥205	¥194	¥253	¥277	¥353	¥430
Otitis Media							
Mild							
Tier 1	¥69	¥117	¥123	¥214	¥246	¥376	¥312
National	¥144	¥114	¥116	¥166	¥167	¥209	¥174
Moderate to Severe							
Tier 1	¥2269	¥3221	¥3050	¥3769	¥3143	¥4768	¥3569
National	¥2269	¥3221	¥3050	¥3769	¥3143	¥4768	¥3569
Source: CHIRA 2013–2015							

**Table 3:** Base Case Results—Total Cases and Costs with and without the PCV13 Program.

	TIER-1 CITIES											
	Beijing			Shanghai			Shenzhen					
	VACC.	NO VACC.	INCR.	VACC.	NO VACC.	INCR.	VACC.	NO VACC.	INCR.			
**Cases of Pneumococcal Disease ^1^**												
Pneumococcal Bacteremia	0.86	1.18	−0.33	1.16	1.61	−0.45	0.43	0.65	−0.22			
Pneumococcal Meningitis	0.23	0.33	−0.10	0.31	0.44	−0.13	0.13	0.20	−0.07			
Pneumonia (Inpatient)	248.80	283.21	−34.41	326.51	361.70	−35.19	136.24	153.68	−17.44			
Pneumonia (Outpatient)	345.32	350.96	−5.63	423.44	428.60	−5.16	167.06	169.60	−2.54			
Otitis Media (Mild)	69.28	72.83	−3.55	63.47	66.72	−3.25	31.22	32.82	−1.60			
Otitis Media (Mod./Sev.)	19.82	22.13	−2.31	18.16	20.28	−2.12	8.93	9.97	−1.04			
**Total Cases**	**684.31**	**730.64**	**−46.33**	**833.05**	**879.35**	**−46.29**	**344.01**	**366.92**	**−22.91**			
**Total Deaths**	**12.74**	**13.56**	**−0.82**	**17.27**	**18.32**	**−1.05**	**4.30**	**4.75**	**−0.45**			
**Total QALYs Lost**	**135.95**	**147.96**	**−12.01**	**181.87**	**196.75**	**−14.88**	**66.20**	**73.86**	**−7.66**			
**Costs ^2^**												
Vaccine Cost	¥331.06	¥0.00	¥331.06	¥295.11	¥0.00	¥295.11	¥149.19	¥0.0	¥149.19			
Pneumococcal Bacteremia	¥1.01	¥1.30	−¥0.30	¥1.36	¥1.74	−¥0.38	¥0.30	¥0.45	−¥0.15			
Pneumococcal Meningitis	¥0.96	¥1.16	−¥0.19	¥1.29	¥1.55	−¥0.26	¥0.28	¥0.37	−¥0.09			
Pneumonia (Inpatient)	¥3706.33	¥3979.43	−¥273.11	¥4966.81	¥5282.32	−¥315.51	¥1461.90	¥1598.34	−¥136.44			
Pneumonia (Outpatient)	¥140.62	¥141.93	−¥1.31	¥179.26	¥180.46	−¥1.20	¥59.07	¥59.66	−¥0.59			
Otitis Media (Mild)	¥46.40	¥46.75	−¥0.35	¥58.79	¥59.10	−¥0.32	¥20.94	¥21.10	−¥0.16			
Otitis Media (Mod./Sev.)	¥157.75	¥164.40	−¥6.65	¥188.84	¥194.93	−¥6.09	¥76.32	¥79.32	−¥3.00			
**Total Cost**	**¥4384.13**	**¥4334.97**	**¥49.16**	**¥5691.46**	**¥5720.11**	**−¥28.65**	**¥1768.01**	**¥1759.24**	**¥8.76**			
**ICER**			**¥4093**			**DOMINANT**			**¥1145**			
	**Other Cities**											
	**Chengdu**			**Karamay**			**Qingdao**				**Suzhou**	
	**VACC.**	**NO VACC.**	**INCR.**	**VACC.**	**NO VACC.**	**INCR.**	**VACC.**	**NO VACC.**	**INCR.**	**VACC.**	**NO VACC.**	**INCR.**
**Cases of Pneumococcal Disease ^1^**												
Pneumococcal Bacteremia	0.80	1.13	−0.34	0.02	0.03	−0.01	0.43	0.62	−0.19	0.51	0.73	−0.22
Pneumococcal Meningitis	0.21	0.32	−0.10	0.01	0.01	0.00	0.12	0.17	−0.06	0.14	0.21	−0.07
Pneumonia (Inpatient)	241.40	266.76	−25.36	6.73	7.54	−0.81	136.13	152.15	−16.02	152.21	168.37	−16.16
Pneumonia (Outpatient)	304.27	308.25	−3.98	8.63	8.77	−0.14	176.60	179.28	−2.68	190.24	192.62	−2.38
Otitis Media (Mild)	49.00	51.51	−2.51	1.69	1.78	−0.09	32.94	34.63	−1.69	29.29	30.79	−1.50
Otitis Media (Mod./Sev.)	14.02	15.65	−1.63	0.48	0.54	−0.06	9.43	10.52	−1.10	8.38	9.36	−0.98
**Total Cases**	**609.69**	**643.61**	**−33.92**	**17.56**	**18.67**	**−1.10**	**355.65**	**377.37**	**−21.72**	**380.78**	**402.08**	**−21.30**
**Total Deaths**	**11.16**	**11.84**	**−0.68**	**0.26**	**0.28**	**−0.02**	**6.14**	**6.51**	**−0.38**	**7.01**	**7.47**	**−0.46**
**Total QALYs Lost**	**119.53**	**129.24**	**−9.71**	**3.03**	**3.30**	**−0.27**	**65.12**	**70.55**	**−5.43**	**78.79**	**85.66**	**−6.87**
**Costs ^2^**												
Vaccine Cost	¥235.76	¥0.00	¥235.76	¥7.80	¥0.00	¥7.80	¥159.37	¥0.00	¥159.37	¥139.00	¥0.00	¥139.00
Pneumococcal Bacteremia	¥0.89	¥1.17	−¥0.28	¥0.02	¥0.03	−¥0.01	¥0.50	¥0.65	−¥0.16	¥0.54	¥0.72	−¥0.17
Pneumococcal Meningitis	¥1.82	¥2.71	−¥0.89	¥0.05	¥0.07	−¥0.03	¥0.99	¥1.49	−¥0.50	¥1.23	¥1.82	−¥0.59
Pneumonia (Inpatient)	¥1358.27	¥1473.49	−¥115.21	¥34.69	¥38.19	−¥3.50	¥763.06	¥834.15	−¥71.09	¥844.59	¥918.43	−¥73.84
Pneumonia (Outpatient)	¥82.22	¥83.02	−¥0.80	¥2.20	¥2.23	−¥0.03	¥47.23	¥47.77	−¥0.54	¥51.01	¥51.49	−¥0.48
Otitis Media (Mild)	¥29.69	¥30.00	−¥0.32	¥0.85	¥0.86	−¥0.01	¥16.96	¥17.17	−¥0.21	¥18.94	¥19.13	−¥0.19
Otitis Media (Mod./Sev.)	¥135.57	¥140.27	−¥4.70	¥3.97	¥4.13	−¥0.16	¥79.35	¥82.51	−¥3.16	¥84.39	¥87.20	−¥2.81
**Total Cost**	**¥1844.21**	**¥1730.66**	**¥113.55**	**¥49.58**	**¥45.52**	**¥4.06**	**¥1067.45**	**¥983.74**	**¥83.71**	**¥1139.71**	**¥1078.79**	**¥60.92**
**ICER**			**¥11,693**			**¥15,132**			**¥15,422**			**¥8862**

Abbreviations: Vacc. = Vaccination; No Vacc. = No Vaccination; Incr. = Incremental; QALYs = Quality-Adjusted Life-Years; IPD = Invasive Pneumococcal Disease; Mod. = Moderate; Sev. = Severe; DE = Direct Effect; IDE = Indirect Effect. Notes: ^1^ Cases in Thousands; ^2^ Costs in Millions of CNY

**Table 4:** Base Case Results—Analysis of Dosing Schedule.

Cities	Vaccination Rate	Price per Dose	Service/Transplantation FEE/DOSE	Vaccination Doses	ICER	GDP per Capita	ICER/GDP	Cost-Effective?
Beijing	70%	¥698	¥25	4	¥4093	¥164,000(2019)	0.025	Yes
				3	Dominant		-----	Yes
Shanghai	70%	¥698	¥5.5	4	Dominant	¥157,300 (2019)	-----	Yes
				3	Dominant		-----	Yes
Shenzhen	70%	¥698	¥25	4	¥1145	¥203,489(2019)	0.006	Yes
				3	Dominant		-----	Yes
Chengdu	70%	¥698	¥30	4	¥11,693	¥103,386 (2019)	0.113	Yes
				3	¥5624		0.054	Yes
Karamay	70%	¥698	¥0	4	¥15,132	¥145,798 (2017)	0.104	Yes
				3	¥7870		0.054	Yes
Qingdao	70%	¥698	¥34	4	¥15,422	¥124,282 (2019)	0.124	Yes
				3	¥8082		0.065	Yes
Suzhou	70%	¥698	¥20	4	¥8862	¥174,129 (2018)	0.051	Yes
				3	¥3807		0.022	Yes

**Table 5:** DE-Only Scenario Results—Total Cases and Costs with and without the PCV13 Program.

	TIER-1 Cities											
	Beijing			Shanghai			Shenzhen					
	VACC.	NO VACC.	INCR.	VACC.	NO VACC.	INCR.	VACC.	NO VACC.	INCR.			
**Cases of Pneumococcal Disease ^1^**												
Pneumococcal Bacteremia	1.16	1.18	−0.03	1.59	1.61	−0.02	0.64	0.65	−0.01			
Pneumococcal Meningitis	0.32	0.33	−0.01	0.43	0.44	−0.01	0.19	0.20	0.00			
Pneumonia (Inpatient)	268.70	283.21	−14.51	348.41	361.70	−13.29	147.14	153.68	−6.54			
Pneumonia (Outpatient)	345.32	350.96	−5.63	423.44	428.60	−5.16	167.06	169.60	−2.54			
Otitis Media (Mild)	69.28	72.83	−3.55	63.47	66.72	−3.25	31.22	32.82	−1.60			
Otitis Media (Mod./Sev.)	19.82	22.13	−2.31	18.16	20.28	−2.12	8.93	9.97	−1.04			
**Total Cases**	**704.61**	**730.64**	**−26.03**	**855.50**	**879.35**	**−23.85**	**355.19**	**366.92**	**−11.73**			
**Total Deaths**	**13.48**	**13.56**	**−0.08**	**18.25**	**18.32**	**−0.07**	**4.71**	**4.75**	**−0.04**			
**Total QALYs Lost**	**146.37**	**147.96**	**−1.59**	**195.29**	**196.75**	**−1.46**	**73.14**	**73.86**	**−0.72**			
**Costs^2^**												
Vaccine Cost	¥331.06	¥0.00	¥331.06	¥295.11	¥0.00	¥295.11	¥149.19	¥0.0	¥149.19			
Pneumococcal Bacteremia	¥1.26	¥1.30	¥0.05	¥1.70	¥1.74	−¥0.04	¥0.43	¥0.45	−¥0.02			
Pneumococcal Meningitis	¥1.15	¥1.16	¥0.00	¥1.55	¥1.55	¥0.00	¥0.37	¥0.37	¥0.00			
Pneumonia (Inpatient)	¥3913.23	¥3979.43	−¥66.20	¥5221.67	¥5282.32	−¥60.65	¥1568.51	¥1598.34	−¥29.83			
Pneumonia (Outpatient)	¥140.62	¥141.93	−¥1.31	¥179.26	¥180.46	−¥1.20	¥59.07	¥59.66	−¥0.59			
Otitis Media (Mild)	¥46.40	¥46.75	−¥0.35	¥58.79	¥59.10	−¥0.32	¥20.94	¥21.10	−¥0.16			
Otitis Media (Mod./Sev.)	¥157.75	¥164.40	−¥6.65	¥188.84	¥194.93	−¥6.09	¥76.32	¥79.32	−¥3.00			
**Total Cost**	**¥4591.48**	**¥4334.97**	**¥256.51**	**¥5946.91**	**¥5720.11**	**¥226.81**	**¥1874.84**	**¥1759.24**	**¥115.59**			
**ICER**			**¥161,237**			**¥155,625**			**¥161,237**			
	**Other Cities**											
	**Chengdu**			**Karamay**			**Qingdao**			**Suzhou**		
	**VACC.**	**NO VACC.**	**INCR.**	**VACC.**	**NO VACC.**	**INCR.**	**VACC.**	**NO VACC.**	**INCR.**	**VACC.**	**NO VACC.**	**INCR.**
**Cases of Pneumococcal Disease ^1^**												
Pneumococcal Bacteremia	1.11	1.13	−0.02	0.03	0.03	0.00	0.61	0.62	−0.01	0.72	0.73	−0.01
Pneumococcal Meningitis	0.31	0.32	0.00	0.01	0.01	0.00	0.17	0.17	0.00	0.20	0.21	0.00
Pneumonia (Inpatient)	256.49	266.76	−6.54	7.19	7.54	−0.35	145.25	152.15	−6.90	162.24	168.37	−6.13
Pneumonia (Outpatient)	304.27	308.25	−2.54	8.63	8.77	−0.14	176.60	179.28	−2.68	190.24	192.62	−2.38
Otitis Media (Mild)	49.00	51.51	−1.60	1.69	1.78	−0.09	32.94	34.63	−1.69	29.29	30.79	−1.50
Otitis Media (Mod./Sev.)	14.02	15.65	−1.04	0.48	0.54	−0.06	9.43	10.52	−1.10	8.38	9.36	−0.98
**Total Cases**	**625.20**	**643.61**	**−11.73**	**18.03**	**18.67**	**−0.64**	**364.99**	**377.37**	**−12.38**	**391.07**	**402.08**	**−11.01**
**Total Deaths**	**11.79**	**11.84**	**−0.04**	**0.28**	**0.28**	**0.00**	**6.48**	**6.51**	**−0.04**	**7.44**	**7.47**	**−0.03**
**Total QALYs Lost**	**128.11**	**129.24**	**0.72**	**3.26**	**3.30**	**−0.04**	**69.79**	**70.55**	**−0.76**	**84.99**	**85.66**	**−0.67**
**Costs ^2^**												
Vaccine Cost	¥235.76	¥0.00	¥235.76	¥7.80	¥0.00	¥7.80	¥159.37	¥0.00	¥159.37	¥139.00	¥0.00	¥139.00
Pneumococcal Bacteremia	¥1.14	¥1.17	−¥0.03	¥0.03	¥0.03	¥0.00	¥0.63	¥0.65	−¥0.02	¥0.70	¥0.72	−¥0.02
Pneumococcal Meningitis	¥2.61	¥2.71	−¥0.10	¥0.07	¥0.07	¥0.00	¥1.42	¥1.49	−¥0.07	¥1.76	¥1.82	−¥0.06
Pneumonia (Inpatient)	¥1435.34	¥1473.49	−¥38.14	¥36.88	¥38.19	−¥1.32	¥808.51	¥834.15	−¥25.65	¥895.63	¥918.43	−¥22.80
Pneumonia (Outpatient)	¥82.22	¥83.02	−¥0.80	¥2.20	¥2.23	−¥0.03	¥47.23	¥47.77	−¥0.54	¥51.01	¥51.49	−¥0.48
Otitis Media (Mild)	¥29.69	¥30.00	−¥0.32	¥0.85	¥0.86	−¥0.01	¥16.96	¥17.17	−¥0.21	¥18.94	¥19.13	−¥0.19
Otitis Media (Mod./Sev.)	¥135.57	¥140.27	−¥4.70	¥3.97	¥4.13	−¥0.16	¥79.35	¥82.51	−¥3.16	¥84.39	¥87.20	−¥2.81
**Total Cost**	**¥1922.32**	**¥1730.66**	**¥191.65**	**¥51.79**	**¥45.52**	**¥6.28**	**¥1113.46**	**¥983.74**	**¥129.72**	**¥1191.43**	**¥1078.79**	**¥112.64**
**ICER**			**¥170,345**			**¥161,710**			**¥171,496**			**¥167,466**

Abbreviations: Vacc. = Vaccination; No Vacc. = No Vaccination; Incr. = Incremental; QALYs = Quality-Adjusted Life-Years; IPD = Invasive Pneumococcal Disease; Mod. = Moderate; Sev. = Severe; DE = Direct Effect; IDE = Indirect Effect. Notes: ^1^ Cases in Thousands; ^2^ Costs in Millions of CNY.

## Data Availability

Data generated for this project come from proprietary models, which are described in detail in the manuscript.

## Supplementary Materials

The following are available online at https://www.mdpi.com/article/10.3390/vaccines9111368/s1, Table S1: Estimate of Incidence Rates
