# Supplementary material for: The Cost-Effectiveness of 13-Valent Pneumococcal Conjugate Vaccine in Seven Chinese Cities
Source: Vaccines (Basel). 2021 Nov 20;9(11):1368. doi: 10.3390/vaccines9111368 (PMC8624982; doi:10.3390/vaccines9111368)
Supplement: Supplementary file 1 [file vaccines-09-01368-s001.zip › vaccines-1436679-supplementary.pdf]

Table S1. Estimate of Incidence Rates.

**PNEUMOCOCCAL BACTEREMIA**	**0 - < 2**	**2 - 4**	**5 - 17**	**18 - 34**	**35 - 49**	**50 - 64**	**65+**
CHIRA							
Shen et al. 2018 (CHIRA 2011)	2.66	8.35	0.94	2.28	0.32	0.87	0.98
CHIRA 2013-2015	0.14	0.29	0.31	0.55	0.15	0.12	0.16
CHIRA Estimate	*1.40*	*4.32*	*0.63*	*1.42*	*0.24*	*0.50*	*0.57*
NHIRD							
Mo et al. 2016 (NHIRD 2002-2007)	3.81	3.80	0.22	0.18	0.36	0.82	4.47
Zhou et al. 2018 (NHIRD 2007)	10.01	12.50	0.74	0.90	0.99	1.00	9.80
NHIRD Estimate	*6.91*	*8.15*	*0.48*	*0.54*	*0.68*	*0.91*	*7.14*
Chen et al.							
Hu et al. 2014	4.48	5.00	48.81	8.25	8.48	9.21	36.00
Caldwell et al. 2014	4.80	4.70	48.80	16.70	16.70	9.20	36.00
Chen et al. Estimate	*4.64*	*4.85*	*48.81*	*12.48*	*12.59*	*9.21*	*36.00*
Che et al. 2014	*10.20*	*9.47*	-----	-----	-----	-----	-----
Maurer et al. 2016	*1.98*	*1.98*	-----	-----	-----	-----	-----
Pneumococcal Bacteremia Estimate	***5.03***	***5.75***	***16.64***	***4.81***	***4.50***	***3.54***	***14.57***
**PNEUMOCOCCAL MENINGITIS**	**0 - < 2**	**2 - 4**	**5 - 17**	**18 - 34**	**35 - 49**	**50 - 64**	**65+**
CHIRA							
Shen et al. 2018 (CHIRA 2011)	1.25	0.58	1.10	0.64	0.24	0.20	0.10
CHIRA 2013-2015	0.05	0.03	0.07	0.03	0.03	0.02	0.02
CHIRA Estimate	0.65	0.31	0.59	0.34	0.14	0.11	0.06
NHIRD							
Mo et al. 2016 (NHIRD 2002-2007)	1.48	0.53	0.11	0.04	0.04	0.08	0.14
Zhou et al. 2018 (NHIRD 2007)	3.89	1.70	0.36	0.20	0.11	0.10	0.20
NHIRD Estimate	2.69	1.12	0.24	0.12	0.08	0.09	0.17
Chen et al.							
Hu et al. 2014	2.82	0.33	14.58	2.46	2.83	3.41	6.86
Caldwell et al. 2014	2.50	0.60	14.60	5.20	.20	3.40	6.90
Chen et al. Estimate	2.66	0.47	14.59	3.83	4.02	3.41	6.88
Che et al. 2014	5.10	4.73	-----	-----	-----	-----	-----
Maurer et al. 2016	1.33	1.33	-----	-----	-----	-----	-----
Pneumococcal Meningitis Estimate	2.49	1.59	5.14	1.43	1.41	1.20	2.37
**PNEUMONIA - INPATIENT**	**0 - < 2**	**2 - 4**	**5 - 17**	**18 - 34**	**35 - 49**	**50 - 64**	**65+**
CHIRA							
Shen et al. 2018 (CHIRA 2011)	14,857	14,059	13,370	2,193	1,131	1,252	1,894
CHIRA 2013-2015	24,581	25,361	12,683	2,038	1,248	1,189	1,818
CHIRA Estimate	*19,719*	*19,710*	*13,027*	*2,116*	*1,190*	*1,221*	*1,856*

NHIRD							
Mo et al. 2016 (NHIRD 2002-2007)	4,625	3,884	459	104	118	367	2,849
Zhou et al. 2018 (NHIRD 2007)	1,274	1,477	210	115	104	244	2,008
NHIRD Estimate	*2,949*	*2,681*	*334*	*110*	*111*	*305*	*2,428*
LITERATURE							
Hu et al. 2014	4,239	3,830	664	113	215	443	3,700
Caldwell et al. 2014	4,239	3,830	664	164	164	443	3,700
Literature Estimate	*4,239*	*3,830*	*664*	*139*	*190*	*443*	*3,700*
Pneumonia – Inpatient Estimate	***8,969***	***8,740***	***4,675***	***788***	***497***	***656***	***2,661***
**PNEUMONIA - OUTPATIENT**	**0 - < 2**	**2 - 4**	**5 - 17**	**18 - 34**	**35 - 49**	**50 - 64**	**65+**
CHIRA							
Shen et al. 2018 (CHIRA 2011)	2,165	2,238	861	254	220	177	190
CHIRA 2013-2015	970	1,045	425	118	112	99	104
CHIRA Estimate	1,568	1,642	643	186	166	138	147
NHIRD							
Mo et al. 2016 (NHIRD 2002-2007)	31,633	38,109	6,272	1,516	1,814	2,967	10,008
Zhou et al. 2018 (NHIRD 2007)	16,421	21,605	7,020	1,194	501	895	3,317
NHIRD Estimate	24,027	29,857	6,646	1,355	1,158	1,931	6,663
Pneumonia – Outpatient Estimate	***12,797***	***15,749***	***3,644***	***771***	***662***	***1,035***	***3,405***
**OTITIS MEDIA (MILD)**	**0 - < 2**	**2 - 4**	**5 - 17**	**18 - 34**	**35 - 49**	**50 - 64**	**65+**
CHIRA 2013-2015	*2.32*	*29.45*	*24.87*	*5.39*	*5.89*	*3.17*	*1.63*
NHIRD							
Mo et al. 2016 (NHIRD 2002-2007)	15,866	17,279	3,978	1,247	1,528	1,892	2,085
Zhou et al. 2018 (NHIRD 2007)	17,657	13,784	-----	-----	-----	-----	-----
NHIRD Estimate	*16,762*	*15,531*	*3,978*	*1,247*	*1,528*	*1,892*	*2,085*
Shen et al. 2018	*10,678*	*10,678*	-----	-----	-----	-----	-----
Otitis Media (Mild) Estimate	***9,147***	***8,746***	***2,002***	***626***	***767***	***948***	***1,043***
**OTITIS MEDIA (MODERATE/SEVERE)**	**0 - < 2**	**2 - 4**	**5 - 17**	**18 - 34**	**35 - 49**	**50 - 64**	**65+**
CHIRA 2013-2015	*15.53*	*105.07*	*98.43*	*10.62*	*8.39*	*4.99*	*2.19*
NHIRD							
Mo et al. 2016 (NHIRD 2002-2007)	6,894	6,202	889	134	252	343	361
Zhou et al. 2018 (NHIRD 2007)	2,050	1,704	-----	-----	-----	-----	-----
NHIRD Estimate	*4,472*	*3,953*	*889*	*134*	*252*	*343*	*361*
Shen et al. 2018	*3,891*	*3,891*	-----	-----	-----	-----	-----
Otitis Media (Mild) Estimate	***2,793***	***2,650***	***494***	***72***	***130***	***174***	***182***
